# Urinary retention complicated by hematocolpos in an adolescent girl: Case report

**DOI:** 10.1016/j.ijscr.2023.108934

**Published:** 2023-10-17

**Authors:** Noor Al-Buloushi, Shahad AlBusairi, Abdullah Alenezi, Mohammed Zahir

**Affiliations:** a Urology Department, Jaber AlAhmed Hospital, Kuwait; b Kuwait Urology Board, KIMS, Kuwait; c Urology Department, Al Jahra Hospital, Kuwait

**Keywords:** Hematocolpos, Urinary retention, Primary amenorrhea, Urologic emergency, Hematometrocolpos, Case report

## Abstract

**Introduction and importance:**

This case report is intended to present an unusual cause of urinary retention by a congenital vaginal obstruction, such as hematocolpos. Hematocolpos is an obstruction of the menstrual flow due to an anomaly of the genital tract, with imperforate hymen being the most common one.

**Case presentation:**

A 12-year-old female patient, was referred to our emergency department for recurrent abdominal pain lasting approximately 90 days. She presented with intermittent dysuria and supra pubic fullness. Ultrasonography confirmed acute urinary retention and features of hematocolpos [7].

**Clinical discussion:**

High index of suspicion is necessary to diagnose and treat such patients promptly. Patients usually present with cyclic pain, primary amenorrhea, incomplete emptying of urine, chronic constipation, back pain and recurrent urinary tract infections. Our patient presented with acute urinary retention.

**Conclusion:**

This case report highlights the importance of early recognition of hematocolpos as a potential cause of cyclic pain, and urinary symptoms. Prompt and accurate diagnosis with the appropriate surgical managements, to resolve their symptoms with successful outcomes.

## Highlights

• Hematocolpos is relatively uncommon condition leading to devastation outcomes.
• High level of suspension is warranted to diagnose and manage those patients.
• This case report presents a unique instance of hematocolpos in an adolescent girl, emphasizing the diagnostic challenges, clinical management, and successful outcomes.
• Diagnosis is primarily based on clinical presentation and imaging modalities, such as ultrasound and MRI.
• Treatment options include surgical hymenotomy or hymenectomy, aiming a patent vaginal outlet and relieve obstruction.

## 1 Introduction

Hematometrocolpos commonly occurs in two age groups: pubertal and menopausal. In the pubertal age group, congenital anomalies from developmental failure of the Müllerian ducts begin to manifest as pelvi-abdominal pain, and primary amenorrhea caused by hematocolpos, which is defined as fluid accumulation in the cervix [10]. Another entity is Hematometra meaning the accumulation of fluid in the uterine cavity [1]. The main causes of hematometrocolpos during the pubertal age group include imperforate hymen, distal vaginal agenesis, transvaginal septum, and other uterine anomalies. Imperforate hymen results from failure of the endoderm of the lower third of the vagina of the urogenital sinus to completely canalize. The exact embryological origin of imperforate hymen is unknown. The hymen normally perforates during the perinatal period at 22 weeks [10]. The incidence of imperforate hymen is 1 in 1000 female births [6].

The rarity of this condition causes it to be undiagnosed until menarche in many cases. Patients commonly present with abdominal, pelvic, or perineal pain. As well as constipation and lower urinary tract symptoms secondary to mechanical obstruction. Urinary retention in the pediatric age group is considered a rare event making is unlikely to be diagnosed early, thus, delaying its treatment similarly our patient. Regardless of the age group, urine retention is a urologic emergency and must be managed immediately. Our patient presented to our community hospital where she was later diagnosed and managed. This case report has been reported in line with SCARE 2020 criteria [12].

## 2 Case presentation

A 12-year-old young girl, 149 cm in height, weighing 55 kg known to have recurrent urinary tract infection (UTI) since childhood with no investigations done. The patient had a history of recurrent visits to the emergency department 3 months prior to admission. She complained of intermittent dysuria and lower abdominal pain.

She was treated as Urinary tract infection and was discharged home.

The patient had dysfunctional voiding. Her mother reported that she holds her urine for long periods of time, specifically in public areas. She usually double voids to empty her bladder. She presented to the emergency room with 90 days history of intermittent dysuria, lower abdomen and suprapubic cyclic pain for the past 2 months. She denied any trauma, constipation or back pain.

### 2.1 Diagnostic assessment

The patient had an unremarkable antenatal, postnatal history and past medical history. Initially the Patient was tachycardic (110 beats per minute). She was afebrile with a normal blood pressure, oxygen saturation, and respiratory rate. A full examination was showed distended abdomen up to the umbilicus with tenderness mainly in the suprapubic area. Patient's sexual characteristics were of Tanner stage 3.

Pelvic examination was then conducted by labial separation in a supine frog-leg position. A bluish bulging hymen obstructing the urethral meatus was seen that was tender to touch.

Laboratory investigations were done and were mostly unremarkable except white blood cell count (11.300/μL).

Renal function test and urine routine were also unremarkable. Urine culture showed no growth.

A decision was made to perform a bedside ultrasonography that showed bladder filled with anechoic fluid with a dilated vagina filled with low level echoes that could be blood products. A 14 French catheter was inserted and drained 750 ml clear urine which relieved her acute pelvic and abdominal pain.

**Fig. 1:**
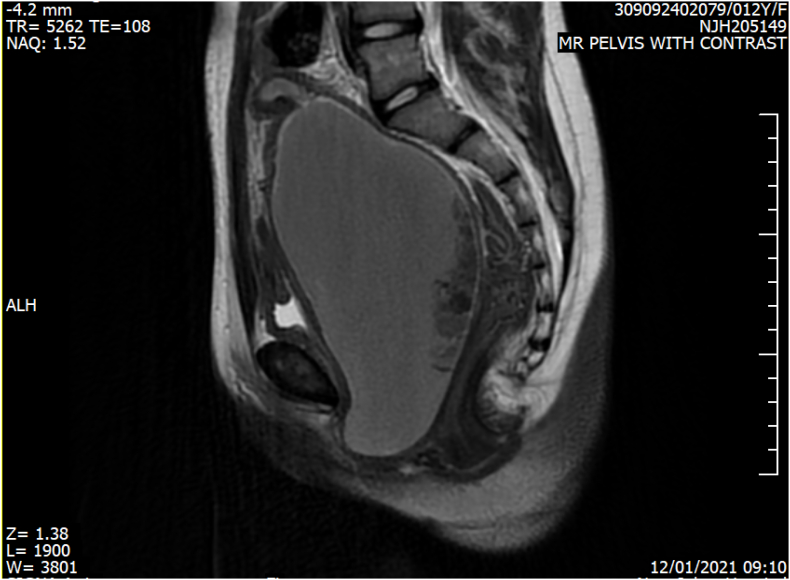
sagittal view MR pelvis with contrast T2 Blood signal contents markedly distended the vagina that extends inferiorly to the level of the perineum, suggesting hematocolpos.

She was then admitted to the urological ward, and an MRI was done (Fig. 1) and revealed a vagina markedly distended with fluid seen along the posterior vaginal wall.

**Fig. 2:**
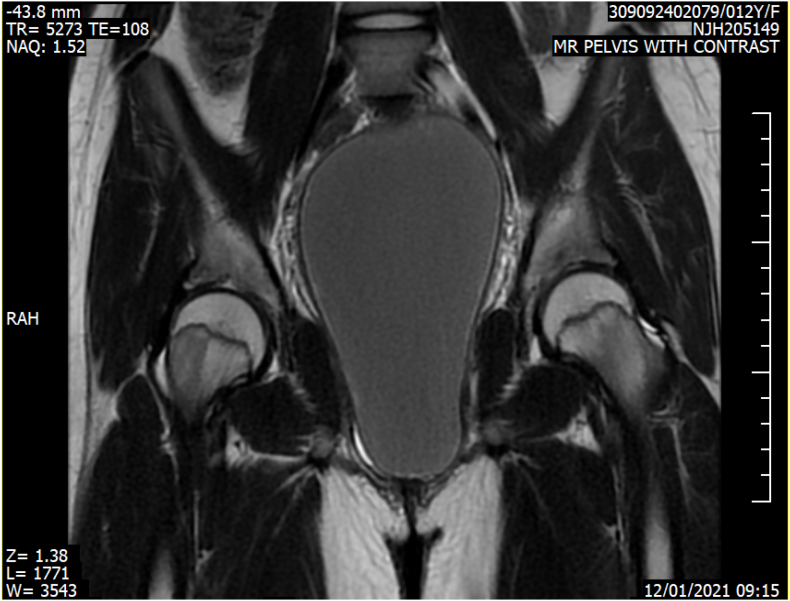
coronal view MR pelvis with contrast T2 showing a distended vagina filled with fluid content T2 hyperintense signal. The bladder is displaced anteriorly.

The uterus was seen displaced superiorly showing its cavity mildly distended with minimal amount of fluid showing signs of hematocolpos (Fig. 2). A multidisciplinary meeting was held with obstetrics and gynecology to discuss the patients' condition. Due to religious reasons no internal examination was conducted and a provisional diagnosis of imperforate hymen of hematocolpos was made.

After counseling the parents about the risks and benefits of hymenotomy, the parents signed a consent forum for surgical drainage.

### 2.2 Therapeutic intervention

An urgent hymenotomy was done on an urgent basis. Two consultants scrubbed in, a urologist as well as a gynecologist both with high level of training. A surgical incision to the hymen was made in the centre of the imperforate hymen which was in a cruciate manner. The hymen was thick and tough which made the incision difficult. Approximately 500 ml of thick tarry colored old menstrual blood with clots were drained with the help of suprapubic manual pressure application. Redundant flaps were later cut, and the edges of the margins were everted by suturing the inner vaginal mucosa to the exterior vestibular mucosa. Vicryl 2–0 braided absorbable sutures were used. The enlargement of the abdomen almost immediately subsided as the vagina emptied.

A medical-legal certificate was given to the family for the medical intervention done causing her loss of virginity.

### 2.3 Follow up

No future surveillance will be needed for this diagnosis. Although she had a 1 week follow up post intervention to check on the wound and proper dressing.

A later follow up was also done with a uroflow and post residual volume to make sure proper voiding has been achieved. An ultrasound was also done to check for vaginal patency. She was also advised to come to the Emergency Room, if she was unable to void, faced intractable pain, hematuria, or fever. The patient had high satisfaction after the surgical intervention which relieved her from a long duration of pelvic pain.

## 3 Clinical discussion

Hematocolpos is a medical condition characterized by the accumulation of menstrual blood in the vagina. It typically results from a blockage in the reproductive tract, such as a vaginal septum or an imperforate hymen [1]. During pubertal period imperforate hymen is the most common cause. Hematocolpos itself does not directly cause urinary retention. Although, in very rare circumstances, may cause urinary symptoms, such as failure to store and failure to empty. It may develop if the collected menstrual blood exerts direct pressure on the adjacent urinary structures, causing difficult voiding. Presentation is usually with pelvic pain, dysuria, lower urinary tract symptoms in the adolescence age group. It is often misdiagnosed as UTI symptoms. Acute urinary retention will result from a delayed diagnosis, as it did for our patient. It is crucial to diagnose adolescent females by evaluating their secondary sexual characteristics and the history of pubertal development.

An imperforate hymen can be diagnosed by clinical examination, showing a tense, bluish bulging membrane. Tumor markers such as CA-125 and CA-19-9 are elevated in imperforate hymen, although these markers are not really necessary for the diagnosis and would delay prompt treatment [11].

The use of ultrasound is usually the preliminary imaging tool in the diagnosis of Müllerian duct anomalies, and adequate enough to detect blood clots in the vagina [10]. However MRI provides more anatomical details like site, thickness of septum with presence or absence of hole/aperture in the septum, but usually not used in an acute setting [4,5]. The decision to undergo surgery is based on multiple factors, including the patient's age, general health, the severity of the obstruction, and the underlying cause of the hematocolpos.

There are several surgical interventions for hematocolpos. One of them is Hymenotomy which is commonly done. An incision is created in the hymen to create an opening to expel the menstrual blood relieving the patient. A special technique such as Z-plasty, may be performed in an attempt to reduce the risk of restenosis [3]. It is done by making a small incision in the upper vagina with diathermy and draining the menstrual blood with laparoscopic suction. Another form of intervention is vaginal septum resection.Vaginal septum is a wall of tissue dividing the vagina. The procedure is done for the patients that have a vaginal septum causing the hematocolpus. The septum will be removed enabling unobstructed flow of menstrual blood from the vagina [3]. A third form of intervention is Vaginoplasty. It may be performed if the patient has a complicated congenital defect of the reproductive system, like vaginal agenesis or vaginal atresia [3,8,9]. A functional vagina is created by the reconstructive surgical surgery known as vaginoplasty. It entails building or enlarging the vaginal canal using already-existing tissues or grafts to enable proper menstruation and sexual function [3,8].

## 4 Conclusion

In summary, hematocolpos is a serious condition that can lead to complications if left untreated. Prompt diagnosis and management are essential to relieve acute symptoms, prevent complications, and restore normal menstrual function. Treatment typically involves a minor surgical procedure to create an opening in the hymen and allow the accumulated menstrual blood to drain. Keeping a high index of suspension is key to diagnosing such rare conditions.

## Consent

A written consent was obtained from the patient and legal guardians for publication of this case report and accompanying images. A copy of the written consent is available for review by the Editor-in-Chief of this Journal in request.

## Ethical approval

Ethical approval is exempted at our institution.

## Funding

No funding sources to be declared.

## Author contribution

Noor Al-Buloushi: literature review, writing, paper editing, manuscript drafting.

Shahad alBusari: literature review, writing.

Abdullah Alenezi: paper editing.

Mohammed Zahir: primary physician, critical review, supervision, final approval.

## Guarantor

Corresponding author: Noor Al-Buloushi.

## Research registration number

1. Researchregistry.

2. researchregistry9374.

3. https://www.researchregistry.com/browse-the-registry#home/.

## Declaration of competing interest

The authors report no declaration of interest.
